# Use of road underpasses by mammals and a monitor lizard in eastern Australia and consideration of the prey‐trap hypothesis

**DOI:** 10.1002/ece3.9075

**Published:** 2022-07-05

**Authors:** Ross L. Goldingay, David Rohweder, Brendan D. Taylor, Jonathan L. Parkyn

**Affiliations:** ^1^ Faculty of Science Southern Cross University Lismore New South Wales Australia; ^2^ Sandpiper Ecological Surveys Pty Ltd Alstonville New South Wales Australia; ^3^ Present address: Jonathan L. Parkyn, NSW Department of Planning, Industry and Environment Australia

**Keywords:** *Aepyprymnus*, *Macropus*, *Notamacropus*, Oxley Highway, Pacific Motorway, *Thylogale*, *Varanus*, *Wallabia*

## Abstract

Road networks continue to expand globally with predictable effects on ecological systems. Research into the effectiveness of road underpasses and overpasses for wildlife has been concentrated in North America and Europe. In Australia, most studies of underpasses have been of relatively short duration and without reference sites to give context to the measured rates of use. We studied 5–7 road underpasses at two locations in eastern Australia over 2–3 years, comparing camera trap detections of animals in underpasses with those at nearby forest sites. Three species of large macropod (wallabies and kangaroos) were frequently detected in the underpasses, with some underpasses traversed 1–4 times per week, and in many cases exceeded detections in the forest. The lace monitor (*Varanus varius*) was detected in all underpasses, often once per week during spring and summer, and infrequently in the forest. At each location, a different small macropod species, including one regionally threatened, showed a higher probability of detection in one underpass compared with several of the forest sites. The vulnerable koala (*Phascolarctos cinereus*) was detected infrequently in underpasses and in the adjoining forest. The short‐beaked echidna (*Tachyglossus aculeatus*) had a high probability of detection in a single underpass. The “prey‐trap hypothesis” postulates that predators will exhibit increased activity at underpasses as a consequence of prey being funneled. We found the red fox (*Vulpes vulpes*) had high activity in some underpasses. However, its activity coincided less than expected with the activity of the mammals most at risk to it. Our results provide no consistent support for the “prey‐trap hypothesis.” Instead, our study confirms the generic value of underpasses for a range of medium‐large mammals as well as one large reptile. Habitat adjoining underpasses exert a strong influence on their use and require greater consideration to maximize underpass use.

## Graphical abstract

We recorded a range of wildlife species in road underpasses at two locations in eastern Australia. Some underpasses were used 1‐4 times per week by wallabies and kangaroos. Habitat adjoining underpasses exerted a strong influence on their use.

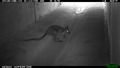

## 1 INTRODUCTION

The impact of roads on wildlife populations is a global concern because these networks occur across most parts of the globe and are increasing in their density and reach (Laurance et al., 2014). Roads lead to direct mortality of animals through vehicle strike and may disrupt wildlife populations in ways that are not easily observed, such as by preventing animals from dispersing across a formerly interconnected landscape (e.g., Olsson et al., 2008; Sawyer et al., 2016). Some wildlife species are at particular risk from these types of disruptions (e.g., species unable to avoid moving vehicles; rare species with low reproductive rates; Fahrig & Rytwinski, 2009) and may require intervention. Government road agencies have responded with greater effort during the last 20 years to reduce the effects of roads on wildlife. The most frequent response has been to install fencing to exclude wildlife from the roadway (Clevenger et al., 2001) and structures to enable animals to cross safely under or over a road (Denneboom et al., 2021).

Road crossing structures for wildlife are intended to serve five objectives: reduce vehicle strike and road‐kill; improve driver safety; enable wildlife to disperse to maintain gene flow; enable seasonal migration; and enable home range movements (Denneboom et al., 2021; Kusak et al., 2009; Sawyer et al., 2012; Simpson et al., 2016; Taylor & Goldingay, 2010). Structures built under the road (underpasses), which includes modified drainage culverts, have been the most widely installed and studied structures (Denneboom et al., 2021; Taylor & Goldingay, 2010). Many studies have been conducted to determine the effectiveness of these structures, with a particular focus on their frequency of use (e.g., Clevenger et al., 2001; Clevenger & Waltho, 2000; Taylor & Goldingay, 2003). Despite reports that underpasses were used by a range of species, there is concern that studies have suffered from design limitations, with many being of relatively short duration and having no control or reference sites to indicate whether the frequency of use of the structures is more or less what could be expected if structures were functioning effectively (van der Grift et al., 2013; van der Ree et al., 2007). Future studies need to respond to these study design issues.

Wildlife underpass studies have been conducted around the world with a much greater number conducted in North America (Taylor & Goldingay, 2010). The relative lack of studies in Australia is concerning because it is regarded as a megadiverse country, with many unique fauna and a disproportionate number of extinctions (Rodrigues et al., 2014). Additional studies of underpasses in Australia will not only benefit wildlife populations in Australia but provide independent assessment of factors identified as influential elsewhere. Thirteen studies in Australia have so far investigated the use or value of underpasses to wildlife populations. While these studies have provided important insights, they have many limitations. Only two studies extended for more than 2 years (Taylor & Goldingay, 2014; van der Ree et al., 2009). Most studies investigated fewer than five underpasses (Bateman et al., 2017; Bond & Jones, 2008; Chachelle et al., 2016; Goosem et al., 2005; Harris et al., 2010; Hayes & Goldingay, 2009; Koehler & Gilmore, 2014; van der Ree et al., 2009) and were focused on a single study area. Only two studies (Chambers & Bencini, 2015; van der Ree et al., 2009) had knowledge of wildlife populations in the habitat surrounding the underpasses, while another two studies radio‐tracked animals to describe their use of the underpasses and the surrounding habitat (Bateman et al., 2017; Chachelle et al., 2016). These limitations and idiosyncrasies mean that the true value of underpasses may be underappreciated and may not lead to improvements in how this mitigation measure is implemented.

The Australian studies to date enable three generalizations about species use of underpasses. Firstly, bandicoots and macropods (kangaroos and wallabies) were regular users of underpasses (Bateman et al., 2017; Bond & Jones, 2008; Chachelle et al., 2016; Chambers & Bencini, 2015; Goosem et al., 2005; Harris et al., 2010; Taylor & Goldingay, 2003). Underpass use by large macropods is particularly important because it will improve road safety for vehicles. Secondly, underpass use was dominated by mammals, reflecting the methods used (sand tracking; wildlife cameras) which favor the detection of medium–large mammals, or because studies targeted mammals by radio‐tracking. Thirdly, studies that demonstrated a benefit to threatened species were highly targeted in location to where those species were abundant (Bateman et al., 2017; Dexter et al., 2016; Harris et al., 2010; van der Ree et al., 2009).

Our study builds on the above generalizations. We use a study design where, rather than attempting to compare crossing rates of the new roads with control or reference roads (e.g., Soanes et al., 2018), we compare traverses past cameras in underpasses with traverses past cameras at randomly selected locations in the adjoining forest (e.g., Andis et al., 2017). This approach overcomes issues relating to whether the target species occur at equal abundance at treatment and reference roads. We studied underpasses below newly constructed highways, commencing approximately 1.5 years after they were open to traffic. This design precludes assessment of the impact of road construction.

A common concern with underpasses is that they may operate as prey traps by allowing predators to focus their foraging to where prey are confined (Hunt et al., 1987; Little et al., 2002). This has been investigated in detail in North America (Ford & Clevenger, 2010; Martinig et al., 2020) and Europe (Mata et al., 2020), but there has been no detailed study in Australia. Red foxes (*Vulpes vulpes*) and feral cats (*Felis catus*) have been implicated in the decline of many small and medium‐sized mammals (Woinarski et al., 2015). Given that these species have often been detected in underpasses in Australia (Bond & Jones, 2008; Chambers & Bencini, 2015; Goosem et al., 2005; Harris et al., 2010), there is a need to investigate whether these predators benefit from the installation of underpasses.

Our study had four aims: (i) to compare detections of different species within underpasses with detections at random sites in the forest, (ii) to identify the species that use underpasses most frequently, (iii) to investigate whether predators use underpasses to trap prey, and (iv) to investigate whether underpasses benefit threatened species. We address these aims with investigations at two locations, approximately 180 km apart, in northeast New South Wales (NSW) in eastern Australia. Including two locations increased the range of species that could potentially use the underpasses and provides a stronger basis for generalization than if conducted at one location. We used the detections to test two competing hypotheses that the detection of species would differ between the underpasses and the forest, due to either avoidance (e.g., forest‐dependent species) or attraction (e.g., predators) to the underpasses, or conversely, that they would use some underpasses more frequently than others. Uneven use of underpasses can arise due to variation in habitat suitability near underpass entrances (e.g., Chambers & Bencini, 2015; McDonald & St Clair, 2004; Ng et al., 2004).

## 2 METHODS

### 2.1 Study areas

**FIGURE 1:**
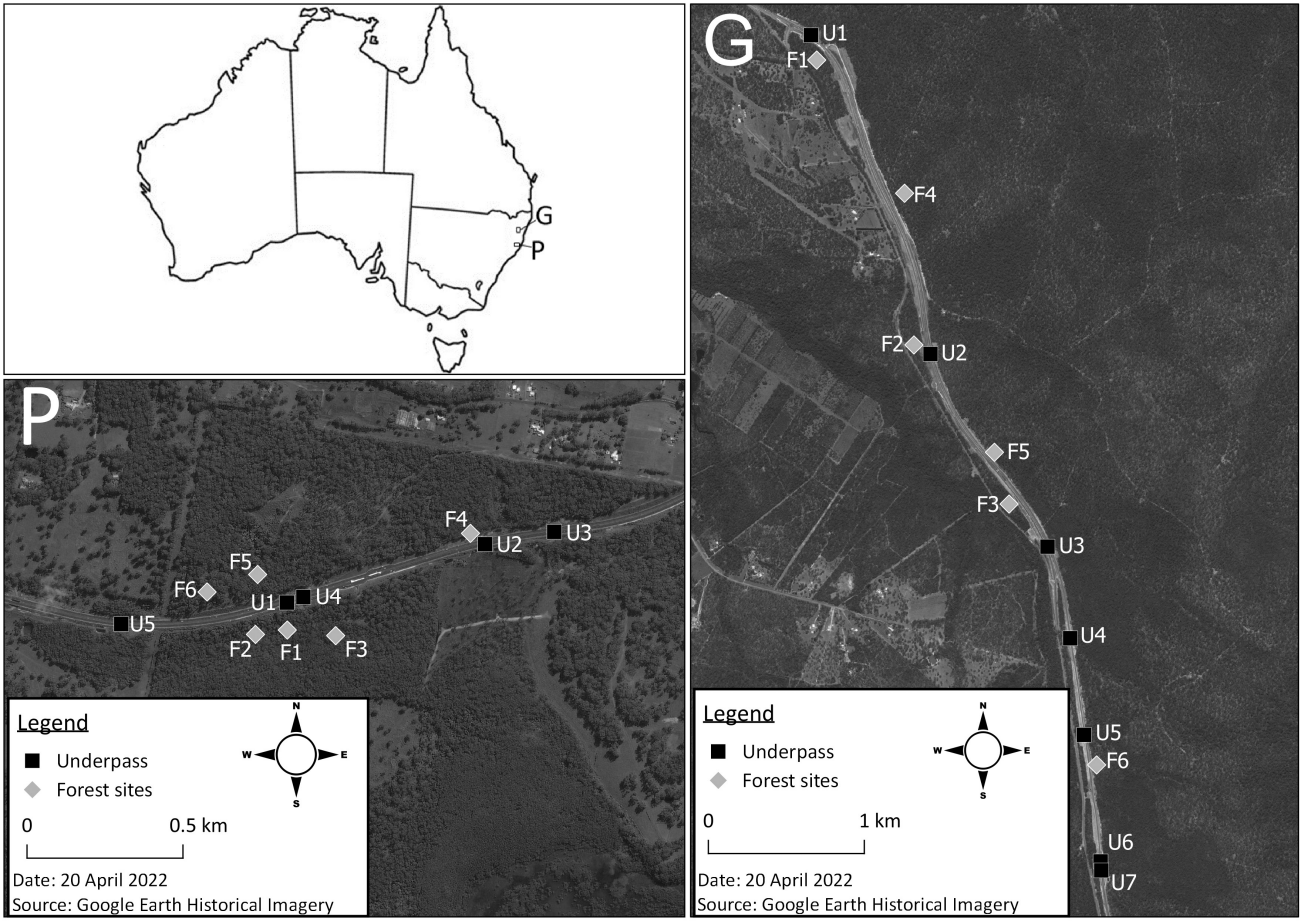
Map of Australia showing the location of the study areas at Grafton (G) and Port Macquarie (P). The aerial images at each location show the location of the underpasses (U) and forest (F) sites

Our study was conducted along the Oxley Highway at Port Macquarie (Port) on the mid‐north coast of NSW and along the Pacific Motorway at Glenugie, 12 km south of Grafton on the north coast of NSW (Figure 1). The Oxley Highway deviation at Port consists of a dual carriageway that extends east for approximately 6 km from the Pacific Motorway. It traverses a large block of open wet sclerophyll forest for a length of approximately 2.2 km. The forest was dominated by tallowwood (*Eucalyptus microcorys*) and blackbutt (*E. pilularis*). There had been no major wildfire since 1994/95, so it contained a very dense understory. Annual rainfall at the nearest weather station averaged 1408 mm (Port airport, #60139; Bureau of Meteorology; www.bom.gov.au). The study area at Glenugie included a 7 km section of new dual carriageway of the Pacific Motorway. It was surrounded on both sides by open dry sclerophyll forest dominated by spotted gum (*Corymbia maculata*) and ironbark (*E. tetrapleura* and *E. fibrosa*), within State Forest that was managed for timber production. The last wildfire in the northern part of the study area occurred in 1994/95 and in the southern part in 2000/01 (NSW Government, 2022). The forest had a mostly open grassy understorey. Annual rainfall averaged 1017 mm (Kangaroo Creek station, 20 km west; #58138; www.bom.gov.au). At both locations, there was a continuous exclusion fence linking all underpasses for the entire length of each study area. This comprised a standard 1.5‐m floppy top fence at Port and 1.2 m stock fence with chicken wire at Grafton. At Port, there were seven pairs of escape ramps, spaced 250–650 m apart, within the exclusion fence (see Goldingay et al., 2018).

**FIGURE 2:**
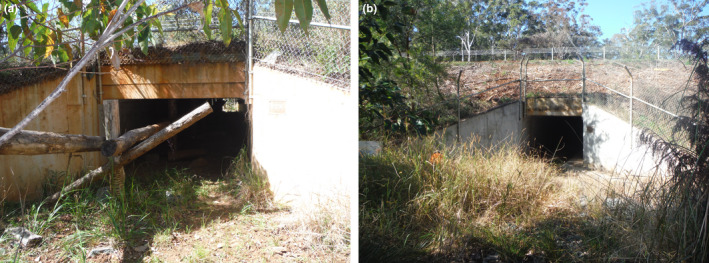
Underpasses at Port Macquarie. (a) The west dedicated fauna underpass with timber railing. (b) A combined fauna underpass

The stretch of highway studied at Port was 24 m wide, consisting of four lanes, with a 6‐m‐wide center median of shrubs (Goldingay, R.L., Rohweder, D., Taylor, B.D, & Parkyn, J.L., unpublished data). Six fauna underpasses were located within the extent of the forest block. Two underpasses were dedicated fauna passages and featured elevated timber railings for arboreal and scansorial species and had earthen floors (Figure 2a). Four underpasses had a concrete floor and functioned as combined drainage and fauna passages (Figure 2b). Five of the underpasses were included in this study (Table 1). The highway was opened to traffic on 7 February 2012. The speed limit on the highway was 90 km/h. Average daily traffic volume in 2015 was 14,300 vehicles per day (RMS, unpublished data).

**TABLE 1:** Details of the different underpasses that were monitored at each location. Distance is measured from either the eastern edge of the forest (Port Macquarie) or the northern end of the study area (Grafton). F = fauna underpass with timber railing, C = combined drainage and fauna function, B = Bebo arch, D = dual cell culvert for drainage and fauna (dimensions shown for one cell)

Location	Code	Distance (m)	Height by width (m)	Length (m)
Port Macquarie	U1‐F	1370	1.8 × 3	32
	U2‐F	715	3 × 3	32
	U3‐C	475	1.8 × 2.4	32
	U4‐C	1315	1.8 × 2.4	32
	U5‐C	1900	1.8 × 2.4	32
Grafton	U1‐B	0	3 × 6	41
	U2‐F	2200	2.4 × 2.4	44
	U3‐D	3600	2.4 × 3	48
	U4‐D	4200	2.1 × 2.4	31
	U5‐C	4800	2.4 × 2.4	21
	U6‐B	5600	3 × 9	33
	U7‐F	5660	2.4 × 2.4	23

**FIGURE 3:**
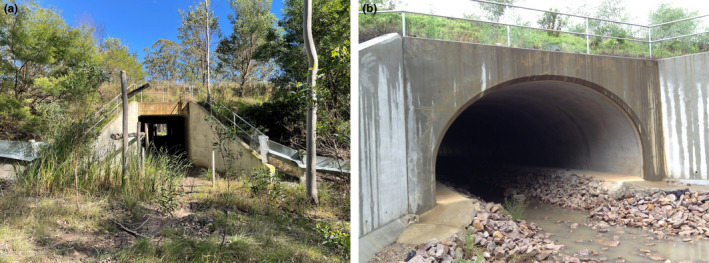
Underpasses at Grafton. (a) A fauna underpass with timber railing. (b) The Bebo arch underpass contained a center drainage area and raised passageways on each side

The stretch of highway studied near Grafton varied in the number of lanes and the separation of northbound and southbound lanes by forest (Goldingay, R.L., Rohweder, D., Taylor, B.D, & Parkyn, J.L., unpublished data). At the time of the study, the new carriageway contained three underpasses (U1–U3) in the northern part and four underpasses (U4–U7) under only the new southbound carriageway in the southern part of the study area (Table 1). In the south, the original highway functioned as the northbound lanes and was separated by 35–55 m of forest from the new southbound carriageway. In the north, the original highway was still in use and was separated from the new freeway by 20–155 m of forest. Underpasses dedicated for fauna passage had soil and mulch floors, and one contained an elevated timber railing (Figure 3a). All other underpasses, including two Bebo Arches (Figure 3b), had concrete floors to function as combined drainage and fauna passages. The highway was 34 m wide and consisted of four lanes, with a 9‐m‐wide grassy median. Following our study, the southern section was converted to a dual carriageway and the old highway was converted to a local road.

### 2.2 Camera monitoring

Two Reconyx HC500 cameras were installed in each end of the seven underpasses (and both cells of the dual cells) at Grafton on February 18–19, 2013. Several cameras were stolen or vandalized in 2014, leading to all cameras being removed on June 18, 2014, and not reinstalled until August 6, 2014. These periods and any malfunctions were treated as missing occasions when detections were collated. Cameras were installed approximately 5 m inside an underpass, on the ceiling, and directed at the entrance. The cameras in three underpasses were repositioned on the underpass wall after 6 months. They were all installed on the walls during 2016/17. Overall, cameras operated in the underpasses for periods of 107–135 weeks. Cameras in the Bebo arches (U1, U6) were positioned on only one of the two passageways so have potentially under‐represented the number of animals traversing the underpass.

Reconyx HC500 cameras were installed in three of the underpasses (U1, U2, and U3) at Port on June 18, 2013, and in a further two underpasses (U4 and U5) on Nov 18, 2015. All cameras operated until Aug 15, 2016. The cameras in U1 were replaced with KeepGuard KG680V camera after 70 weeks due to theft. This camera was of a similar size and could be set up in a similar way to the HC500. There was no evidence that this camera performed differently to the others. Underpasses U2 and U3 were monitored for 165 weeks, U1 for 120 weeks and U4 and U5 for 24–38 weeks. One camera was installed at least 10 m inside both ends of each underpass except for U2, which had a single camera installed in the middle of the underpass. Cameras were installed at 1–1.5 m high on the side wall and angled to detect animals moving along the ground. At both locations, cameras were held in locked security housings.

Reconyx HC500 cameras (without lures) were also installed at six random forest sites at each location (three each side of the freeway) within approximately 100 m of the road and between the most western and eastern underpasses at Port and between the most northern and southern underpasses at Grafton. At Port, four cameras were installed in early October 2014 and operated for 79–98 weeks. Another two cameras were installed in mid‐October 2015 and operated for 32–43 weeks. These cameras operated concurrently with the underpass cameras. At Grafton, all six cameras operated for 76 weeks between November 15, 2015, and May 7, 2017. The timing of installation meant the forest cameras operated concurrently with the underpass cameras at Grafton for only 11 weeks. We have assumed that the periods of monitoring were of sufficient length to characterize weekly detections at each site (most underpasses > 100 weeks; most forest cameras > 70 weeks). Cameras were attached to trees ~50 cm above the ground in small pre‐existing forest clearings. The clearings were at least 3 m deep in front of the camera and 2–3 m wide. Although no attempt was made to equalize the fields of view of the forest and underpass cameras, the width was equivalent. There was no indication that any systematic bias existed. Our objective was to characterize the number of detections past random locations in the forest. Cameras were programmed to record five images when triggered with no delay between triggers. They were set to medium–high sensitivity. These cameras and those in the underpasses were serviced approximately every 6–8 weeks to replace SD cards and batteries. Images were subsequently viewed on a computer and the species present, date and time of each record, and direction of animal movement recorded on a spreadsheet. Only medium–large mammals and a monitor lizard were detected with sufficient frequency to analyze.

The number of passes (i.e., detections) of each species was collated for each camera. A pass in an underpass was defined as movement toward or away from the camera. Occasions where animals had clearly stopped and turned around within an underpass were not included. Successive passes by the same species in an underpass or in the forest were only scored if an arbitrary 30 min had elapsed and the animal had left the field of view or if different individuals were evident (e.g., differences in size or markings). Multiple individuals of the same species were scored if >1 was seen in the same image. The cameras at each end of an underpass did not always record the same passage of an animal. This appeared to reflect the speed of movement of individuals, which was usually much greater than past a camera in the forest. The timing of passes at each end of an underpass was matched, so passes recorded by both cameras were counted as one pass. For each underpass, we summed the number of passes of each species.

### 2.3 Analysis of detection and detection hypotheses

Data were initially collated to show the number of weekly passes by each species in each underpass or at each forest site. The most frequently detected species were analyzed in detail at each location. We pooled the long‐nosed bandicoot (*Perameles nasuta*) and the northern brown bandicoot (*Isoodon macrourus*) into a bandicoot group because these species can sometimes be difficult to distinguish and there were insufficient data to analyze them separately. We chose to adopt an occupancy approach (see MacKenzie et al., 2018) to analyze our data. However, rather than focus on occupancy, which is precluded here because most species were detected across most sites (underpasses and forest), we focused on the probability of detection, which can be viewed as a measure of habitat use. We constructed weekly detection histories of our species, indicating detected (1) or not detected (0) across sites for the total duration of monitoring of underpasses and forest sites. Occupancy modeling can handle missing values (−) which in this case occurred when cameras malfunctioned, were not set properly or were stolen, or when underpass and forest sites were not surveyed concurrently.

For each species, we tested three hypotheses: (i) that the probability of detection differed between the forest and the underpass sites; (ii) that species are patchy in their use of the landscape so that the probability of detection would be high or low (i.e., patchy) at individual sites rather than similar across underpasses or across forest sites; and (iii) that the probability of detection was equivalent across all sites (i.e., a null model).

We used single‐season occupancy modeling implemented within program presence version 12.24 (USGS Patuxent Wildlife Research Centre, Laurel MD, USA). With one exception, we did not examine temporal variation in detection because the timing of forest monitoring was less extensive and not fully aligned with underpass monitoring. The exception was the lace monitor (*Varanus varius*), which was not present during the cooler months of the year. To account for this, we fitted a detection model that included season, which contrasted autumn and winter with spring and summer.

We started our modeling with a null model that estimated detection as equal across sites. We then fitted an “underpass” model, which estimated detection at underpass sites as different to that at forest sites. A model was then fitted to represent the “patchy detection” hypothesis. An initial model was fitted with a different covariate (i.e., dummy variable) for every site. Many sites had similar estimates of detection (overlapping standard errors), so a further model was fitted with a reduced number of site covariates. We compared models using AIC_c_, which is the Akaike Information Criterion corrected for small sample size (Burnham & Anderson, 2004). Models were ranked from lowest to highest AIC_c_. Models where ∆AIC_c_ <2 were considered equally plausible to explain the data. Models where ∆AIC_c_ was >10 had essentially no support to explain the data. For many of the species, the occupancy parameter estimate converged on one so was fixed at 1.0 to ensure model convergence.

### 2.4 Tests of the prey‐trap hypothesis

The predators of interest were the red fox (*Vulpes vulpes*), the feral cat (*Felis catus*), and the dingo/dog (*Canis familiaris*). The lace monitor, a large predatory lizard, was also present but its diurnal activity meant it posed little threat to the mammals using the underpasses. We investigated two prey groups: the bandicoots and small macropods, and the medium‐sized macropods (i.e., wallabies). These groups were relatively abundant in our study areas and are known to be frequent prey of the mammalian predators (Barker et al., 1994; Glen et al., 2006; Lunney et al., 1990; Marlow et al., 2015; Stokeld et al., 2018; Triggs et al., 1984; Woolley et al., 2019).

The fauna underpass prey‐trap hypothesis proposes that underpasses concentrate the movement of fauna to confined and predictable locations that could be readily exploited by predators (Hunt et al., 1987; Little et al., 2002; Martinig et al., 2020). This hypothesis gave rise to a number of predictions that we tested in our study areas: (1) predators should be detected more frequently at underpasses than in the forest, (2) predators should focus their activity at underpasses where potential prey are more frequently detected, and (3) the temporal use of underpasses by predators and potential prey should not be independent. We assumed that predators are highly responsive to prey and that the underpasses are equally accessible to predators and prey. We also assumed that if predators target prey, the detection of predators in underpasses would reflect that rather than predators increasing their activity outside the entrances to the underpasses where they could not be detected. Prediction 1 was tested by comparing the detection models described above. This prediction would be supported if a model that contrasted underpasses with forest sites fit the data better than the patchy or null model and detection was higher in the underpasses compared with the forest. Prediction 2 was tested using a descriptive approach due to the small number of underpasses where predators had high levels of activity. We compared the estimates of the probability of detection of predator and prey for these underpasses. We also compared a sum of the total number of detections of predators and prey in these underpasses. This prediction would be supported if predator activity aligned with underpasses with the highest prey activity.

To test the third prediction, we collated temporal data on underpass use only for those underpasses where both prey and predators were frequently detected. This restriction was imposed so that the outcome was not determined by sparse data. The data collated were the number of nights in which a predator (a), a prey (b), and both predator and prey (c) were detected, and the number of sample nights in the comparison (d = a + b – c). Nights were defined as the period 1700–700 h to encompass the nocturnal and dusk period when the mammalian predator and prey species are predominantly active. This period encompassed 99% of red fox detections (Goldingay, R.L., Rohweder, D., Taylor, B.D, & Parkyn, J.L., unpublished data). Mata et al. (2020) used a daily (i.e., 24‐h) detection record to investigate interactions between predators and prey in underpasses. Although a one‐night window of concurrent detection may appear a coarse measure of interaction, it eliminates some assumptions about predator–prey encounters, such as the length of the interval between prey–predator detections needed to suggest prey are being targeted. In the case of underpasses U1 and U2 at Port, the mean interval between fox and bandicoot or pademelon detection was 193 ± 28 (SE) min and between fox and wallaby detection was 250 ± 26 min.

For each comparison, we calculated the nightly probability that a predator was detected (a/d), and that the prey was detected (b/d) on the sample nights. The product of these (a/d*b/d) gives the expected probability of both being detected on the same night given their activity. A binomial test was applied in IBM SPSS Statistics 25 to test whether the observed proportion of nights when both predator and prey were detected differed to what was expected given their individual probabilities of being detected (i.e., whether their co‐detection was independent). The binomial test calculates a one‐tailed significance probability of obtaining the observed proportion or a more extreme proportion by chance (Zar, 2009).

## 3 RESULTS

### 3.1 Species detected

#### 3.1.1 Port Macquarie

**FIGURE 4:**
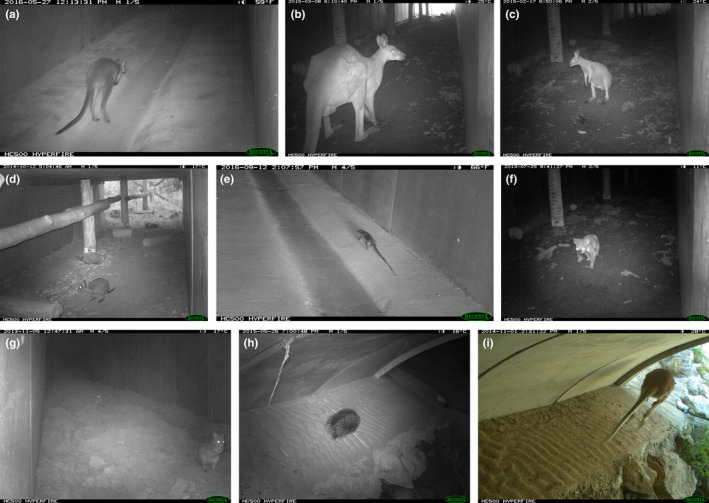
Some of the species recorded in the underpasses. (a) Swamp wallaby, (b) eastern grey kangaroo, (c) red‐necked wallaby, (d) red‐necked pademelon, (e) lace monitor, (f) koala, (g) rufous bettong, (h) echidna, and (i) red‐necked wallaby

**FIGURE 5:**
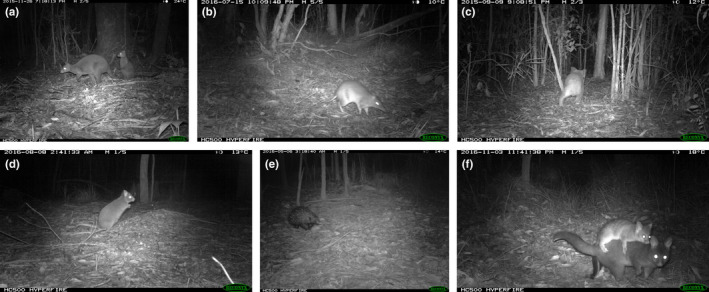
Some of the species recorded at the random forest sites. (a) Red‐necked pademelon, (b) long‐nosed bandicoot, (c) koala, (d) rufous bettong, (e) echidna, and (f) common brushtail possum

Excluding the predator species, there were 3476 detections of native mammals and one reptile in the underpasses at Port (Table 2). The swamp wallaby (*Wallabia bicolor*) (Figure 4a) was detected more than once per week in three underpasses and up to 3.9 times per week in one. It was commonly detected at sites in the forest, including once per week at two sites. The eastern grey kangaroo (*Macropus giganteus*) and the red‐necked wallaby (*Notamacropus rufogriseus*) (Figure 4b,c) were detected more than once per week in one underpass but infrequently in the forest. The red‐necked pademelon (*Thylogale thetis*) (Figure 4d) was detected >2 times per week in one underpass and >3 times per week at one forest site (Figure 5a). The pooled bandicoot group (Figure 5b) was detected just under once per week in one underpass and at one forest site. The lace monitor was detected in three underpasses more than once per week (Figure 4e) and much less commonly in the forest. The koala (Figure 4f), the only threatened species recorded in the underpasses at Port, was detected infrequently in the underpasses or in the forest (Figure 5c). The red fox was the most commonly detected predator, using one underpass more than once per week (Table 2). Only a single feral cat was detected across all sites.

**TABLE 2:** Number of passes (detections) per 52 weeks by species detected in the underpasses (U) and in the forest (F). At Grafton U3 and U4 contained dual cells in the same structure. Those designated “b” were not used in the data analysis. Shading highlights the site for each species with the highest number of detections

Species	F1	F2	F3	F4	F5	F6	U1	U2	U3	U4	U5	U6	U7	U3b	U4b
Port Macquarie															
Swamp wallaby	27.3	57.9	38.3	29.4	42.3	58.0	89.5	201.1	105.3	21.7	21.9	–	–	–	–
Eastern grey kangaroo	0.6	2.0	0.0	13.6	0	0	7.6	117.2	12.6	19.5	0	–	–	–	–
Red‐necked wallaby	0	0	0.0	3.8	0	0	2.2	76.6	2.8	0	0	–	–	–	–
Bandicoots	48.8	38.4	10.9	15.8	9.8	33.9	14.5	51.1	5.0	0	1.4	–	–	–	–
Red‐necked pademelon	48.8	174.9	18.6	9.0	9.8	19.3	139.0	7.6	1.6	0	0	–	–	–	–
Lace monitor	1.9	10.4	4.4	0.8	3.3	4.8	36.2	65.2	79.7	99.7	23.3	–	–	–	–
Red fox	0	0	0	0.8	1.6	1.2	12.3	35.0	86.4	0	0	–	–	–	–
Feral cat	0	0	0	0	0	0	0	0.3	0	0	0	–	–	–	–
Dog/dingo	4.4	7.8	1.6	19.6	1.6	2.4	0	0.6	0.6	0	0	–	–	–	–
Brushtail possums	31.1	118.3	6.0	3.8	1.6	6.0	2.5	1.6	0	0	19.2	–	–	–	–
Koala	1.9	3.3	1.1	0.8	0	0	0.9	0.3	2.2	0	0	–	–	–	–
Echidna	0.6	0.7	0.0	0.8	0	0	0.3	1.3	0	0	0	–	–	–	–
Fallow deer	10.8	5.2	12.6	16.6	6.5	0	0	1.3	0	0	0	–	–	–	–
Grafton															
Swamp wallaby	0	0	11.6	0	0.6	0.7	0	1.2	0	0	4.9	0.4	0.5	0	0
Eastern grey kangaroo	17.8	10.9	0	0	1.1	0	5.4	5.7	0.9	0	1.6	0	0	0	0
Red‐necked wallaby	21.9	35.6	0	2.6	5.0	1.4	11.1	14.2	0	0.4	15.0	8.3	10.3	2.9	1.2
Whiptail wallaby	6.8	0	0	0	0	0	2.9	0	0	0	0	0	0	0	0
Bandicoots	22.6	25.3	59.5	10.6	24.9	14.4	7.0	9.3	1.9	0.4	0.4	22.5	0.5	21.0	0.4
Rufous bettong	0	9.6	0.7	8.8	0.6	0	0	43.1	0	0	0	0	0.5	0	0
Lace monitor	2.1	2.7	2.7	0	0	0.7	71.4	67.4	5.6	10.0	79.0	77.4	13.0	12.0	30.8
Red fox	0	1.4	0	0	1.7	0.7	5.0	13.8	0	0.4	2.0	0.8	0.5	1.0	1.5
Feral cat	2.1	0	1.4	0	0	0	7.8	0.8	0	0.9	0.4	1.7	0	0	0
Dog/dingo	0.7	6.2	1.4	0.9	6.1	0.7	1.7	2.8	0.5	1.8	8.1	6.2	1.2	1.0	3.1
Brushtail possums	23.3	11.6	0	4.4	2.8	0	0	2.0	0	0	0	0	0	0	0
Echidna	8.9	3.4	1.4	0	0.6	0	16.5	2.0	0	0	0	0.8	0	0.4	0.4
Feral horse	0	0	0	39.7	8.3	1.4	0	0	0	0	0	0	0	0	0

#### 3.1.2 Grafton

Excluding the predatory species, there were 1408 detections of native mammals and one reptile in the underpasses at Grafton (Table 2). The rufous bettong (*Aepyprymnus rufescens*) (Figures 4g and 5d), a regionally threatened species, was detected in one underpass almost once per week, which was more frequent than its detection at any of the forest sites. The lace monitor was detected in all underpasses and in four more than once per week. Its use of the underpasses greatly exceeded its detection at the forest sites. The echidna (Figures 4h and 5e) was detected in one underpass at a higher rate than at any of the forest sites. The bandicoots were detected in two underpasses at levels greater than or equivalent to that at five of the forest sites. The larger macropods (Figure 4i) were detected in many of the underpasses but at a lower frequency compared with the forest. The koala was detected only once in an underpass. Common brushtail possums (*Trichosurus vulpecula*) (Figure 5f) were detected at four of the forest sites but only in one underpass. Water rats (*Hydromys chrysogaster*) were detected on five occasions in the underpasses but never in the forest. The three mammalian predators were detected in most underpasses but at a relatively low frequency of mostly <10 times per year (Table 2). The fox and feral cat were rarely detected in the forest, whereas the dingo was detected at the forest and underpass sites at an equivalent frequency.

### 3.2 Probability of detection at Port Macquarie and Grafton

**FIGURE 6:**
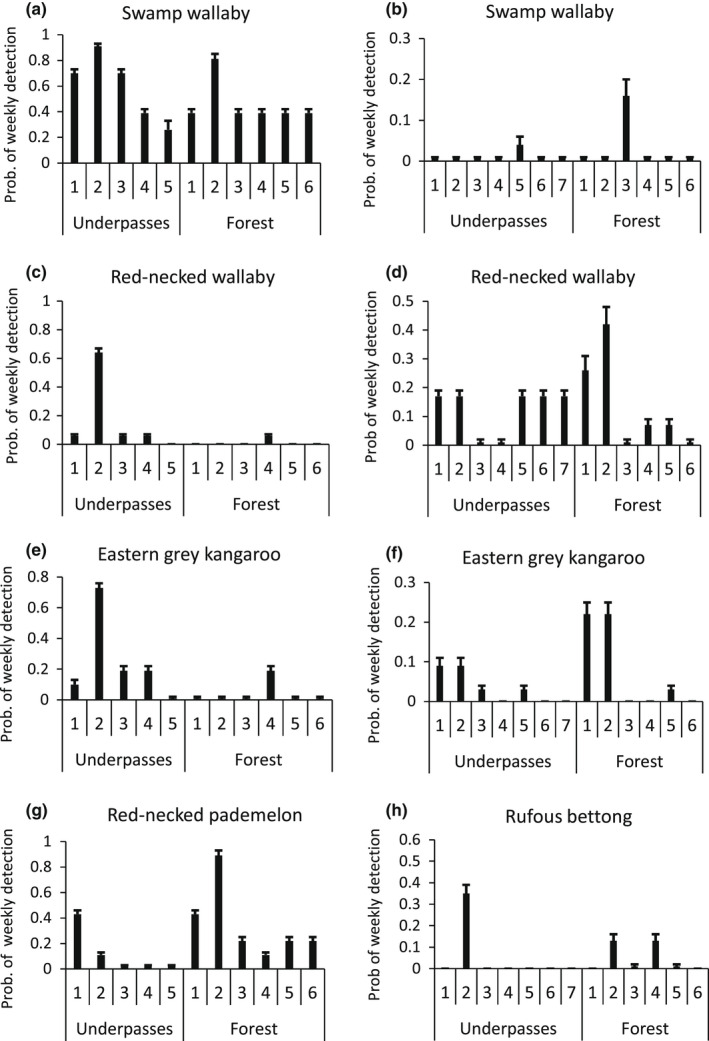
Probability of weekly detection (mean + SE) of different macropod species within underpasses and at random forest sites at Port Macquarie (left panels) and Grafton (right panels). Note the *y*‐axis scale differs across panels

**FIGURE 7:**
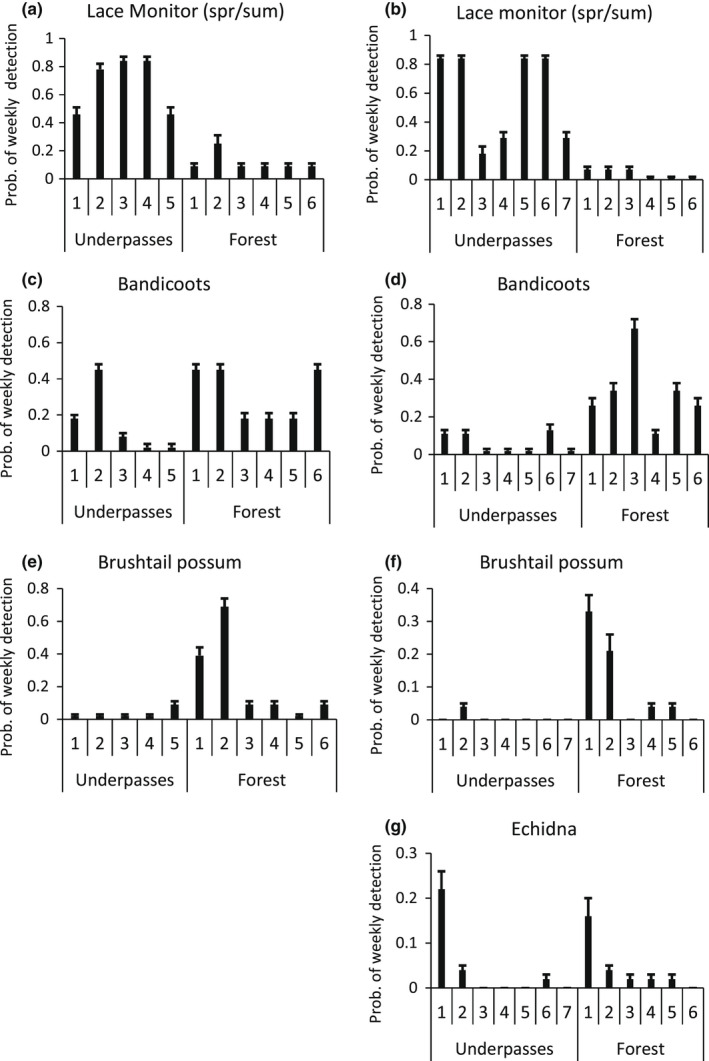
Probability of weekly detection (mean + SE) within underpasses and at forest sites of other species at Port Macquarie (left panels) and Grafton (right panels). Values for the lace monitor are for the spring–summer season only. Note the *y*‐axis scale differs across panels

There was no support for a null model or a model contrasting underpass and forest sites for any species at either Port (Table 3) or Grafton (Table 4). In every case, a model which allowed detection to differ among a reduced set of sites had the greatest support. These models revealed that for many species, the probability of detection (hereafter detection) in at least one underpass exceeded detection at several of the forest sites (Figures 6 and 7). Detection of the swamp wallaby at Port was very high in three of the underpasses (≥0.7) and exceeded that at five of the forest sites (Figure 6a). It was infrequently detected at Grafton (Figure 6b). Detection of the red‐necked wallaby at Port was very high in one underpass (0.66) but negligible at all other sites (Figure 6c). In contrast, it was detected with a probability of about 0.2 per week in many underpasses which exceeded that at four of the forest sites at Grafton (Figure 6d). Detection of the eastern grey kangaroo at Port was very high in one underpass (>0.7) and exceeded that at all forest sites (Figure 6e). It was detected infrequently in the underpasses at Grafton and less frequently at four of the forest sites (Figure 6f). The small macropods were detected moderately frequently (>0.3 per week) in specific underpasses, as well as in the forest suggesting they were influenced by habitat heterogeneity (Figure 6g,h).

**TABLE 3:** Model selection results of weekly detection at Port Macquarie at underpass and forest sites. Variables in the models conditioned detection on whether a site was an underpass or forest (underpass) that some sites were different and some were equivalent (patchy), or sites were all equivalent (null). For the lace monitor, “season” contrasts spring and summer with autumn and winter. *W*—model weight; *K*—number of parameters

Model	AIC_c_	∆AIC_c_	*W*	*K*
Swamp wallaby				
p(Patchy)	1097.30	0.00	1.00	6
p(Underpass)	1205.85	108.55	0.00	3
p(null)	1257.60	160.30	0.00	2
Eastern grey kangaroo				
p(Patchy)	607.95	0.00	1.00	5
p(Underpass)	793.56	185.61	0.00	3
p(null)	857.60	249.65	0.00	2
Red‐necked pademelon				
p(Patchy)	790.38	0.00	1.00	6
p(Underpass)	989.18	198.80	0.00	3
p(null)	1037.39	247.01	0.00	2
Lace monitor				
p(Patchy+season)	691.36	0.00	1.00	7
p(season)	894.53	203.17	0.00	3
p(Patchy)	911.52	220.16	0.00	6
p(Underpass)	932.30	240.94	0.00	3
p(null)	1067.06	375.70	0.00	2
Red‐necked wallaby				
p(Patchy)	413.05	0.00	1.00	4
p(Underpass)	605.61	192.56	0.00	3
p(null)	616.41	203.36	0.00	2
Bandicoots				
p(Patchy)	956.07	0.00	1.00	5
p(Underpass)	1067.28	111.21	0.00	3
p(null)	1069.98	113.91	0.00	2
Fox				
p(Patchy)	617.74	0.00	1.00	5
p(Underpass)	667.23	49.49	0.00	3
p(null)	720.88	103.14	0.00	2
Brushtail possum				
p(Patchy)	466.89	0.00	0.95	5
p(Underpass)	584.97	118.08	0.00	3
p(null)	668.96	202.07	0.00	2
Dingo/dog				
p(Patchy)	257.33	0.00	0.98	4
p(Underpass)	265.03	7.70	0.02	3
p(null)	299.37	35.44	0.00	2

**TABLE 4:** Model selection results of weekly detection at Grafton at underpass and forest sites. Variables in the models conditioned detection on whether a site was an underpass or forest (underpass) that some sites were different and some were equivalent (patchy), or sites were all equivalent (null). The lace monitor also included a “season” covariate. *W—*model weight; *K*—number of parameters

Model	AIC_c_	∆AIC_c_	*W*	*K*
Swamp wallaby				
p(Patchy)	206.09	0.00	1.00	4
p(Underpass)	228.80	22.71	0.00	3
p(null)	243.96	37.87	0.00	2
Eastern grey kangaroo				
p(Patchy)	396.58	0.00	1.00	5
p(Underpass)	430.15	33.57	0.00	3
p(null)	441.13	44.55	0.00	2
Rufous bettong				
p(Patchy)	328.27	0.00	1.00	5
p(Underpass)	406.27	78.00	0.00	3
p(null)	421.28	93.01	0.00	2
Lace monitor				
p(Patchy+season)	802.54	0.00	1.00	7
p(Patchy)	1107.44	304.90	0.00	6
p(season)	1167.08	364.54	0.00	3
p(Underpass)	1226.30	423.76	0.00	3
p(null)	1393.84	591.30	0.00	2
Red‐necked wallaby				
p(Patchy)	888.22	0.00	1.00	6
p(null)	967.56	79.34	0.00	2
p(Underpass)	969.84	81.62	0.00	3
Bandicoots				
p(Patchy)	867.82	0.00	1.00	7
p(Underpass)	948.02	80.20	0.00	3
p(null)	1116.55	248.73	0.00	2
Fox				
p(Patchy)	274.52	0.00	1.00	5
p(Underpass)	302.64	28.12	0.00	3
p(null)	304.94	30.42	0.00	2
Dingo				
p(Patchy)	403.56	0.00	1.00	4
p(Underpass)	415.27	11.71	0.00	2
p(null)	418.71	15.15	0.00	3
Echidna				
p(Patchy)	359.86	0.00	1.00	6
p(Underpass)	414.53	54.67	0.00	3
p(null)	415.01	55.15	0.00	2
Common brushtail possum				
p(Patchy)	278.39	0.00	1.00	5
p(Underpass)	321.90	43.51	0.00	3
p(null)	332.86	54.47	0.00	2
Feral cat				
p(Patchy)	216.43	0.00	1.00	4
p(Underpass)	243.96	27.53	0.00	2
p(null)	245.30	28.87	0.00	3

The lace monitor provided a consistent result across the two locations, being detected at a very high rate in over half the underpasses (Figure 7a,b). The bandicoots showed a varied response, with detection in most underpasses (Figure 7c,d) but higher detection overall in the forest. The brushtail possum, encompassing the mountain brushtail (*Trichosurus caninus*) at Port and common brushtail at Grafton, showed a very different response to most other species. It was detected infrequently in the underpasses but at high levels at some forest sites (Figure 7e,f). The echidna was detected infrequently at Port (Table 2) but more frequently at Grafton, including in one underpass (Figure 7g).

### 3.3 Tests of the prey‐trap hypothesis

#### 3.3.1 Predators should be detected more frequently at underpasses than in the forest

**FIGURE 8:**
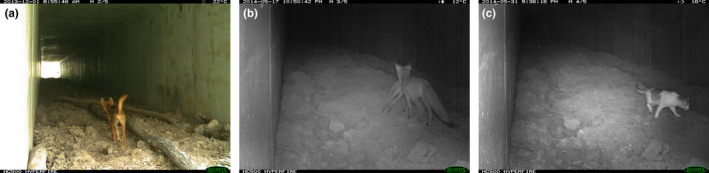
Images of the predators detected in the underpasses. (a) Dingo, (b) red fox with bettong prey, and (c) feral cat

**FIGURE 9:**
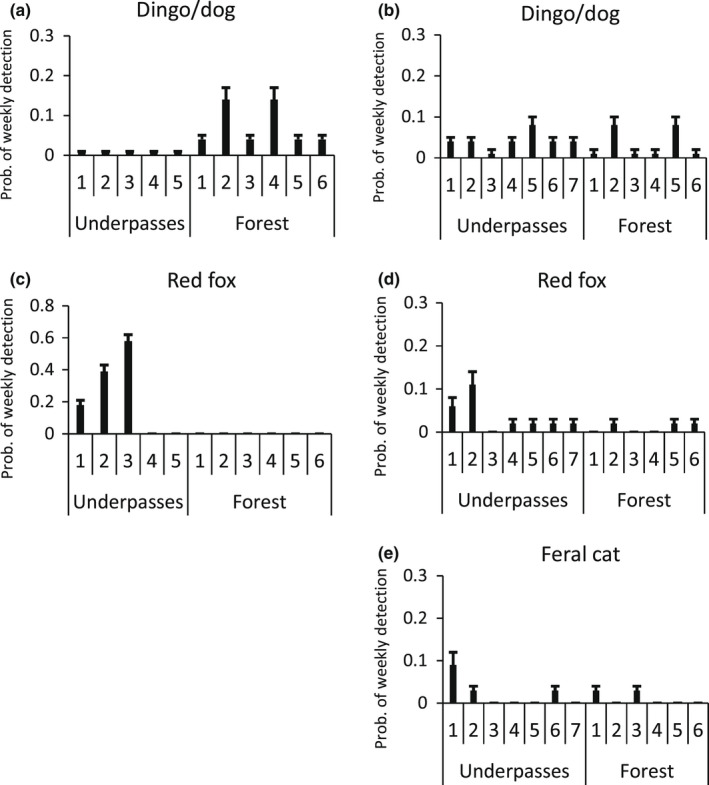
Probability of weekly detection (mean + SE) of predators within underpasses and at forest sites at Port Macquarie (left panels) and Grafton (right panels)

In all cases, the patchy site detection model showed the best fit to the predator data (Tables 2 and 3). The dingo/dog (Figure 8a) was detected more frequently in the forest, especially at two sites, than in the underpasses at Port, allowing the patchy model to be favored (Figure 9a). The dingo/dog had a low overall probability of detection at Grafton with greater variation observed in the forest. The red fox (Figure 8b) was only detected at three of the underpasses at Port and not in the forest (Figure 9c). Its apparent absence at two underpasses led to the patchy model being favored. At Grafton, its probability of detection was low overall but higher at two of the underpasses (Figure 9c,d). The feral cat (Figure 8c) was detected only once at Port and infrequently at Grafton (Figure 9e). The overall findings provide only partial support for the first prey‐trap prediction that detection would be higher at the underpasses, and only in relation to the fox. Given the dingo and feral cat were detected infrequently, they have not been considered in the other predictions.

#### 3.3.2 Predators should focus their activity at underpasses where potential prey are more frequently detected

At Port, small macropods had a very high probability of detection at U1 (Figure 6g), and bandicoots and medium‐sized macropods had a very high probability of detection at U2 (Figure 6a,c). The fox had its highest probability of detection at U3 (Figure 9c). Although the swamp wallaby also had a high probability of detection at U3, other potential prey had low levels of detection at U3. Summing the weekly activity (Table 2) of potential prey (swamp wallaby, red‐necked wallaby, bandicoots, pademelon) showed that activity of these prey in U2 (336 detections per year [D/Y]) was 2.9 times higher than their activity in U3 (115 D/Y) and their activity in U1 was 2.1 times higher than in U3. This suggests that foxes did not respond as predicted to underpasses with the highest prey activity.

At Grafton, the red fox was most likely to be detected (Figure 9) and had its highest level of activity in U2 (Table 2). This underpass had the highest level of prey activity (68 D/Y), primarily due to the rufuous bettong, and one detection was of a fox carrying a bettong (Figure 8b). However, foxes were rarely detected in other underpasses (U5, U6, and U7) where prey activity was high, though 30–46% that of U2. Therefore, although foxes were detected where prey abundance was highest, there appeared to be little targeting of other underpasses.

#### 3.3.3 The temporal use of underpasses by predators and potential prey should show a significant association if predators align their activity to exploit prey using the underpasses

Sufficient data were available to conduct six tests encompassing three underpasses at Port and one underpass at Grafton (Table 5). The prey group involving the smaller species was dominated by pademelons in one underpass and bandicoots in another. At Grafton, this group was dominated by bettongs. In all seven tests, involving the two different‐sized prey groups, the observed proportion of nights in which both predators and prey were detected was significantly lower than the proportion expected. This result enables the prediction to be rejected.

**TABLE 5:** Results of binomial tests comparing the expected and observed proportion of nights when both predators (red foxes) and prey were detected in different underpasses. *N* = number of sample nights; Signif. = probability of detecting observed or more extreme value

Location/underpass	Prey	*n*	Expected	Observed	Signif.
Port‐U1	Bandicoots/pademelons	245	0.280	0.074	.001
Port‐U1	Wallabies	517	0.179	0.077	.001
Port‐U2	Pademelons/bandicoots	219	0.157	0.081	.001
Port‐U2	Wallabies	222	0.171	0.077	.001
Port‐U3	Wallabies	358	0.303	0.115	.001
Grafton‐U2	Bettongs/bandicoots	142	0.179	0.021	.001
Grafton‐U2	Wallabies	67	0.257	0.015	.001

## 4 DISCUSSION

### 4.1 Use of underpasses

Our study has provided a more detailed examination of underpass use by Australian wildlife than any previous study. It was conducted at two widely spaced locations, extended for 2–3 years, compared underpass detections with those at random forest sites, and evaluated use by several threatened species. Our study is not without its own limitations, such as insufficient replication of different types of underpass, which precluded investigating whether underpass attributes influenced use. However, it was sufficiently comprehensive to enable several important findings: (1) macropods were frequent users of the underpasses, as reported in previous studies; (2) bandicoots showed frequent use of few underpasses, despite frequent detection in the adjoining forest; (3) a large reptile, the lace monitor, showed very high use of many of the underpasses; and (4) the red fox was the only predator that used the underpasses with high frequency, enabling a detailed examination of the prey‐trap hypothesis (see below).

The differences among species in the use of the underpasses at the two locations highlight the important role that the adjoining habitat can play in underpass use. The small macropods illustrate this point. The species differed across the two locations but both showed frequent use of one underpass, with detections exceeding that at many of the forest sites. These species, the red‐necked pademelon and the rufous bettong, have not been documented using underpasses previously. However, this finding is not surprising given that regular use of some underpasses by small macropods has been documented before (Bateman et al., 2017). One interesting finding not documented previously was the frequent use of an underpass by the echidna. Overall, these findings confirm the generic value of underpasses and that the more underpasses and locations that are studied the more species that will be detected using underpasses.

### 4.2 Detections in underpasses versus in the forest

We detected five species of macropod, two species of bandicoot, the echidna and a monitor lizard using some of the underpasses with high frequency (>0.5 times per week). Our monitoring within the adjoining forest revealed that the probability of detection in the underpasses often exceeded that at some of the forest sites, suggesting the underpasses were favored for use, and also that some of the variation in use is a consequence of habitat heterogeneity. There was no indication that camera placement led to systematically reduced detections in the forest because all species showed variation in detection across the underpasses as well as across the forest, and some species had very high detection at some forest cameras compared with many of the underpasses. The exclusion fencing that was present may also have led to animals being funneled. Andis et al. (2017) adopted a similar approach of comparing detections in underpasses with those in the adjoining habitat. They highlighted the value of this control‐impact design to control for spatial variability in detections. They found that deer and carnivores were detected more often moving through the underpasses compared with the adjoining habitat and that there was substantial variation across individual structures. They found only one species, the coyote, to be detected less often in underpasses compared with the natural habitat. We found the brushtail possum, which encompassed two species, was detected infrequently in the underpasses whereas it was detected at a high frequency at some of the forest sites. This is consistent with the observation of infrequent use of underpasses by these species in earlier studies (Bond & Jones, 2008; Chambers & Bencini, 2015; Taylor & Goldingay, 2003). This requires further investigation to understand why brushtail possums may be reluctant to use these underpasses. However, road crossing structures are not simply intended to allow foraging movements on either side of a road but also need to enable dispersal movements and gene flow across a landscape, and therefore infrequent use (i.e., detections) may still indicate an effective structure.

Given the importance that adjacent habitat can play in underpass use (see Clevenger & Waltho, 2000; McDonald & St Clair, 2004; Ng et al., 2004), the question arises whether underpasses should be more purposely located or whether habitat restoration at underpass entrances should be more purposeful. Currently in eastern Australia, habitat restoration near underpasses is generally minimal and mostly to do with erosion control. Consideration has been given to provide structures within underpasses (e.g., Goldingay et al., 2019; Goosem et al., 2005; Mansergh & Scotts, 1989), but much less attention has been given to restoring habitat outside underpasses to cater for the needs of different species. The many underpasses installed during the last 10 years as part of the Pacific Highway upgrade project in eastern Australia provide an opportunity for replicated field experiments to investigate the role of adjacent habitat.

### 4.3 Threatened species

In Australia, wildlife road crossing structures are often installed for threatened species (e.g., Mansergh & Scotts, 1989). In eastern Australia, concern about the impacts of new roads on the threatened koala has been responsible for the installation of vast lengths of road‐side fencing and large numbers of underpasses (e.g., Lunney et al., 2022; Taylor & Goldingay, 2003). Our study provides some insight into the effectiveness of this strategy for the koala and other threatened species. We found koalas used underpasses very infrequently, but detections were also infrequent in the adjoining forest. At Port, the koala population was reduced after road construction because, prior to road clearing, nine koalas were captured and translocated 7.5 km away (Phillips, 2018). Dexter et al. (2016) radio‐tracked 58 koalas near roads with exclusion fences in southeast Queensland and found that only 15 made road crossings and confirmed that use of underpasses was uncommon.

Our study detected other threatened species using the underpasses. The regionally threatened rufous bettong was detected frequently in one underpass at Grafton, despite relatively infrequent detections in the adjoining forest. We detected the regionally threatened brush‐tailed phascogale (*Phascogale tapoatafa*) once in an underpass but also only once in the forest. The endangered spotted‐tailed quoll (*Dasyurus maculatus*) was detected once in the forest at each study location but never in an underpass. The wide range of species detected in the underpasses suggests threatened species will benefit. These species usually occur at low density, so it is expected they would be detected infrequently within an underpass. The conclusion we draw is that underpasses are likely to assist in facilitating gene flow for many different threatened species and, therefore, assist their conservation.

### 4.4 Tests of the prey‐trap hypothesis

Our study provides the first comprehensive investigation of the prey‐trap hypothesis for wildlife underpasses in Australia. Previous studies in Australia present either anecdotal or circumstantial accounts (Harris et al., 2010; Hunt et al., 1987). Our analysis, which involved multiple underpasses monitored over >2 years in two independent landscapes, and several species that are commonly preyed on by predators, suggests these underpasses did not act as prey traps. That does not mean that predators at individual underpasses elsewhere will not have an adverse impact on prey species (e.g., Harris et al., 2010). The main prey species in the present study were macropods, which have an ability to move rapidly through open habitats, so may not be at any greater risk to predators in underpasses.

We made several predictions from the prey‐trap hypothesis including that predators would be detected more often in the underpasses than in the forest. Only the red fox was consistently detected more frequently in underpasses compared with the forest. We predicted predators would target underpasses where potential prey were most common. There was only partial support for this. At Port, the red fox showed its highest level of activity in U3 (with a concrete floor) but activity of potential prey was 2.9 times higher in U2. At Grafton, the red fox had its highest level of activity in U2 (with an earthen floor), which had the highest level of prey activity, but foxes were rarely detected in other underpasses where prey activity was relatively high. Underpass features such as floor type do not seem to be influential. These observations suggest foxes may have been influenced by landscape features rather than prey activity alone.

The most definitive test of the prey‐trap hypothesis tested the prediction that predators should align the timing of their use of underpasses to match that of their prey. We conducted seven tests of this prediction using data from different underpasses and involving different prey groups. The central tenet of this assessment is that if predators and prey use the same underpass independently of each other, the proportion of nights when they are both detected will reflect the proportion of nights they individually use the underpass. Deviation from that may reflect either avoidance by the prey or targeting by the predator. In every case, we found that the proportion of nights when both predators (red foxes) and prey were detected in underpasses was significantly lower than expected. This potentially suggests that prey show some avoidance of the predator. A limitation of this analysis is that we assumed that detection of foxes within an underpass reflects their hunting intention. It is possible that they may have hunted immediately outside an underpass where they could not be detected by our cameras. However, Harris et al. (2010) found a significant correlation between daily red fox activity and bandicoot activity within an underpass, and subsequently bandicoots disappeared from the underpass. Future studies could also place cameras immediately surrounding underpass entrances to test the above assumption.

Our study builds on evidence from other systems, which provide limited support for the prey‐trap hypothesis. Ford and Clevenger (2010) used camera detections of ungulates and carnivores in Canada to compare the intervals between prey–predator sequences and predator–prey sequences. They predicted that if predators actively pursue prey the prey–predator interval should be shorter than the predator–prey interval, whereas the converse would indicate avoidance of underpasses by prey due to predator activity. If predators and prey use underpasses independently, then these intervals should be about equal. Their data supported the notion that predators and prey used the underpasses independently. Martinig et al. (2020) tested camera‐detection sequences through underpasses of small mammal prey and predators and found shorter intervals and greater frequency of prey–prey sequences compared with prey–predator sequences, which was contrary to their predictions based on the prey‐trap hypothesis. They suggested deviation from predictions in their Canadian system may arise because prey species were more abundant in the surrounding landscape compared with the predators. Mata et al. (2020) used co‐occurrence modeling to analyze data from tracking strips at underpasses and overpasses in Spain for three prey groups (mouse‐sized up to lagomorphs) and five predator types (mustelids up to large canids). They found evidence of avoidance of predators by some prey as well as some positive associations, suggesting predators targeted their prey. This varied result may suggest differences in morphology and ecology play a role, and that it is too simplistic to expect a single response across all predator–prey pairs. Caldwell and Klip (2020) found evidence in underpasses in California of prey avoidance of predators as well as predators favoring locations and times of higher prey activity.

These findings suggest that prey may be very responsive to their predators, and to use underpasses where complex habitats are not available may require predator avoidance. The small number of detailed studies into the prey‐trap hypothesis suggests this hypothesis is not universally applicable. Some prey groups may be more vulnerable to predators than others, such as those that rely on habitat cover to avoid predators (e.g., McDonald & St Clair, 2004). These studies provide evidence that prey may exhibit avoidance behavior to reduce their risk. This is not surprising given that predators will provide scent cues to which prey can respond (see Kats & Dill, 1998). Further study of the prey‐trap hypothesis is warranted. Providing habitat complexity within underpasses (e.g., refuges) is a management response that may alleviate the risk of predation for some species.

## AUTHOR CONTRIBUTIONS

**Ross L. Goldingay:** Conceptualization (equal); data curation (equal); formal analysis (lead); funding acquisition (equal); investigation (equal); methodology (equal); project administration (equal); resources (equal); software (lead); supervision (equal); validation (lead); visualization (lead); writing – original draft (lead); writing – review and editing (lead). **David Rohweder:** Conceptualization (equal); data curation (equal); funding acquisition (equal); investigation (equal); methodology (equal); project administration (equal); resources (equal); supervision (equal); validation (equal); writing – review and editing (supporting). **Brendan D. Taylor:** Data curation (equal); investigation (equal); methodology (equal); project administration (equal); validation (equal); writing – review and editing (supporting). **Jonathan L. Parkyn:** Investigation (equal); writing – review and editing (supporting).

## CONFLICT OF INTEREST

The authors have no conflicts of interest to declare.

## Data Availability

Data used for analysis in this study are accessible at Dryad: https://doi.org/10.5061/dryad.cvdncjt6m.
